# Deciphering the Active Ingredients and Molecular Mechanisms of *Tripterygium hypoglaucum* (Levl.) Hutch against Rheumatoid Arthritis Based on Network Pharmacology

**DOI:** 10.1155/2020/2361865

**Published:** 2020-01-09

**Authors:** Yunbin Jiang, Mei Zhong, Fei Long, Rongping Yang

**Affiliations:** ^1^College of Pharmaceutical Sciences and Chinese Medicine, Southwest University, Chongqing 400715, China; ^2^College of Pharmacy, Chengdu University of Traditional Chinese Medicine, Chengdu 611137, China

## Abstract

*Tripterygium hypoglaucum* (Levl.) Hutch (THH) shows well clinical effect on rheumatoid arthritis (RA), but the active ingredients and molecular mechanisms remain unclear. This work was designed to explore these issues by network pharmacology. Compounds from THH were gathered by retrieving literatures. Compound-related and RA-related genes were identified using databases, and the overlapping genes were identified by Venn diagram. The active ingredients and genes of THH against RA were confirmed by dissecting interactions between overlapping genes and compounds using Cytoscape. SystemsDock website was used to further verify the combining degree of key genes with active ingredients. Pathway enrichment analysis was performed to decipher the mechanisms of THH against RA by Database for Annotation, Visualization and Integrated Discovery. A total of 123 compounds were collected, and 110 compounds-related and 1871 RA-related genes were identified, including 64 overlapping genes. The target genes and active ingredients of THH against RA comprised 64 genes and 17 compounds, the focus of which was PTGS2, triptolide, and celastrol. SystemsDock website indicated that the combing degree of PTGS2 with triptolide or celastrol was very good. The mechanisms of THH against RA were linked to 31 signaling pathways, and the key mechanism was related to inhibition of inflammation response through inactivating TNF and NF-kappa B signaling pathways. This work firstly explored the active ingredients and mechanisms of THH against RA by network pharmacology and provided evidence to support clinical effects of THH on RA.

## 1. Introduction

*Tripterygium hypoglaucum* (Levl.) Hutch (THH), a traditional Chinese medicine (TCM), has been used to treat systemic lupus erythematous and rheumatoid arthritis (RA) in China for over 60 years [1]. Chinese Pharmacopoeia indicates that THH patent medicine (Kunming Shanhaitang tablet) is a legally licensed drug in China. In recent years, the therapeutic effect of THH on RA has aroused the attention of clinicians and basic researchers. Clinical study indicated that the combined application of THH and low-dose methotrexate showed well clinical effect and safety on elderly onset RA [2]. Basic study suggested that THH exhibited well therapeutic effect on rat with RA by reducing arthritis index, joint swelling, and controlling the balance of cytokines level, such as IL-1*β*, TNF-*α*, and TNF-*β* [3, 4]. A study found that THH inhibited proliferation and induced apoptosis of synovial cells from patients with RA, but showed almost no impact on proliferation and apoptosis of normal synovial cells [5]. These basic research studies provided scientific evidence to support the clinical application of THH in treating RA, but the molecular mechanisms of THH against RA are still unclear. Meanwhile, there is no study to decipher the active ingredients of THH against RA. Therefore, study on the active ingredients and molecular mechanisms of THH against RA needs to be strengthened.

Holistic theory is the central rule of TCM treatments of various diseases, and to provide more scientific evidence to support the therapeutic effect of THH on RA, investigation of the active ingredients and molecular mechanisms of THH against RA should reflect the TCM holistic theory. Network pharmacology is a systematic analytical technology used to explore the interaction network of multiple factors such as diseases, drugs, genes, and protein targets [6]. Network pharmacology can explore the active ingredients and molecular mechanisms of TCM with a holistic perspective by emphasizing the paradigm shift from “one target, one drug” to “network target, multicomponent therapeutics” [7, 8]. Hence, network pharmacology is a feasible method to investigate TCM-related issues with holistic perspective. In recent years, network pharmacology has been widely used to decipher the active ingredients and molecular mechanisms of TCM [9–11].

**Figure 1:**
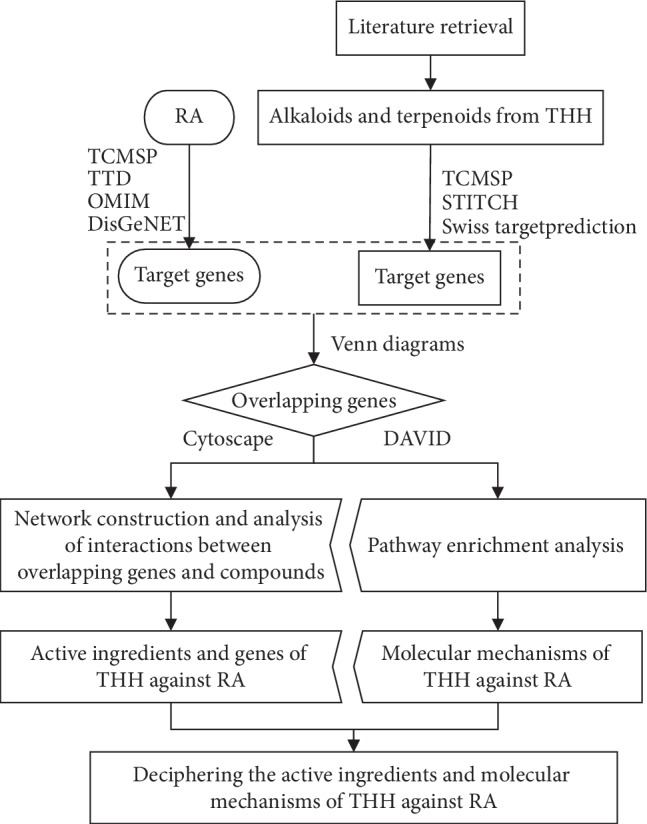
Workflow of network pharmacology analysis.

In the present study, network pharmacology was used to decipher the active ingredients and molecular mechanisms of THH against RA. The workflow is as follows (Figure 1). First, alkaloids and terpenoids from THH were identified by retrieving literatures, and genes related to these compounds were identified using public databases. Then, RA-related genes were identified using public databases, and the overlapping genes between compounds and RA target genes were obtained. Third, the key active ingredients and genes of THH against RA were identified by dissecting the interactions between overlapping genes and compounds. Last, pathway enrichment analysis for overlapping genes was performed to decipher the molecular mechanisms of THH against RA.

## 2. Materials and Methods

### 2.1. Alkaloids and Terpenoids Database Construction and Identification of Their Target Genes

Compounds in THH consist of alkaloids, terpenoids, flavonoids, steroids, tannins, and glucides, but alkaloids and terpenoids are its main active ingredients group [12]. Therefore, the names or structures of alkaloids and terpenoids in THH were collected by retrieving literature studies in this work, and their molecular formulas and SMILES were identified by PubChem (https://pubchem.ncbi.nlm.nih.gov/), SciFinder (https://scifinder.cas.org/), or Molget (http://www.molget.com/).

Target genes related to all alkaloids and terpenoids were gathered from Traditional Chinese Medicine Systems Pharmacology Database and Analysis Platform (TCMSP, http://lsp.nwu.edu.cn/tcmsp.php) [13] and predicted using STITCH (http://stitch.embl.de/) and SwissTargetPrediction (http://swisstargetprediction.ch/) with the “Homo sapiens” setting [14, 15]. To obtain more credible compounds target genes, compound with the highest “Tanimoto score” was selected to predict the genes of target compound, which were further filtered by setting “minimum required interaction score” as “high confidence (0.700)” in STITCH. Meanwhile, the predicted compounds-related genes in SwissTargetPrediction were screened by setting the threshold value of “gene probability” as >0.6.

### 2.2. Identification of RA Target Genes

RA target genes were identified by retrieving 4 public databases [16], including TCMSP, Therapeutic Target Database (TTD, http://bidd.nus.edu.sg/group/cjttd/) [17], Online Mendelian Inheritance in Man (OMIM, https://omim.org/), and DisGeNET (http://www.disgenet.org/). When retrieving the 4 databases, “rheumatoid arthritis” was selected as the search term, and all retrieved genes were defined as RA target genes. The overlapping genes between compounds target genes and RA target genes were identified and visualized by Venn diagramand plotted using the OmicShare tools, a free online platform for data analysis (https://www.omicshare.com/tools).

### 2.3. Network Construction and Analysis of Interactions between Overlapping Genes and Compounds

The results of TCMSP retrieval, STITCH prediction, and SwissTargetPrediction prediction were used to identify the interactions between overlapping genes and compounds. Cytoscape ver 3.7.1 (https://cytoscape.org/) was used to construct, visualize, and analyse the network of interactions between overlapping genes and compounds. In the network, nodes represented genes and compounds, and edges indicated interactions between genes and compounds. Degree value of genes or compounds represented the edges numbers of genes or compounds in the network and was used to identify the importance of compounds and genes in THH against RA. The bigger the degree value of compounds or genes were, the more important compounds or genes were in THH against RA, suggesting that these compounds or genes were key active ingredients and genes of THH against RA. Additionally, SystemsDock (http://systemsdock.unit.oist.jp/iddp/home/index) was used to further verify the combining degree of key genes with active ingredients [18]. It is generally believed that a Docking Score value above 4.25 indicates that there is a certain binding activity between the ingredient and target, above 5.0 shows that the ingredient has a good binding activity with target, and above 7.0 suggests that the ingredient has a strong binding activity with target [19].

### 2.4. Pathway Enrichment Analysis for Overlapping Genes

Pathway enrichment analysis for overlapping genes was performed using Database for Annotation, Visualization and Integrated Discovery ver. 6.8 (DAVID, https://david.ncifcrf.gov/) with the “Homo sapiens” setting. The results of Kyoto Encyclopedia of Genes and Genomes (KEGG) pathway enrichment were used to explore the molecular mechanisms of THH against RA. Bubble chart of the concerned KEGG pathways was plotted using the OmicShare tools.

## 3. Results

### 3.1. Alkaloids and Terpenoids Database and Target Genes

Alkaloids and terpenoids database of THH were constructed by retrieving literatures and consisted of 123 alkaloids and terpenoids (Supplementary [Supplementary-material supplementary-material-1]). The results of TCMSP retrieval, STITCH prediction, and SwissTargetPrediction prediction (Supplementary [Supplementary-material supplementary-material-1]) suggested that a total of 110 genes linked to 18 compounds from 123 alkaloids and terpenoids, including 280 interactions, were identified, and no genes were related to another 105 compounds.

### 3.2. Identification of RA Target Genes

**Figure 2:**
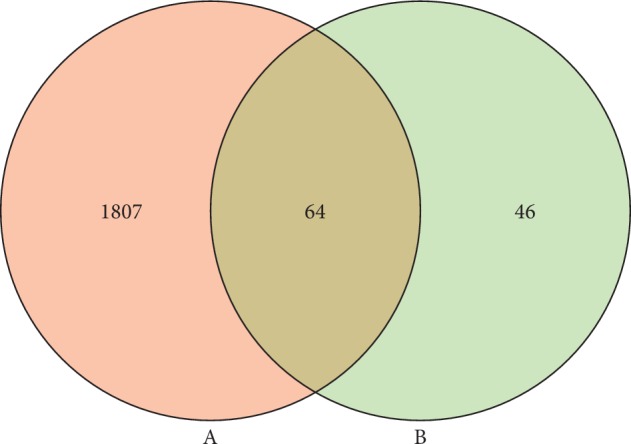
Sixty-four overlapping genes between RA-related 1871 target genes (A) and compounds-related 110 target genes (B).

The results of TCMSP, TTD, OMIM, and DisGeNET databases retrieval indicated that 1871 genes were related to RA, and the information is listed in Supplementary [Supplementary-material supplementary-material-1]. As shown in Figure 2, the Venn diagram showed that 64 overlapping genes were identified by matching the compound-related 110 target genes with RA-related 1871 target genes.

### 3.3. Active Ingredients and Genes of THH against RA

**Figure 3:**
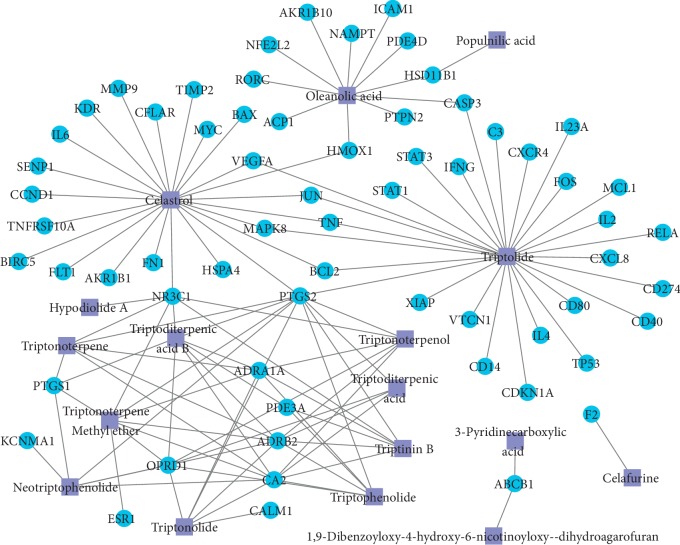
Network with 81 nodes and 122 edges linking RA-related 64 target genes and 17 compounds from THH.

**Figure 4:**
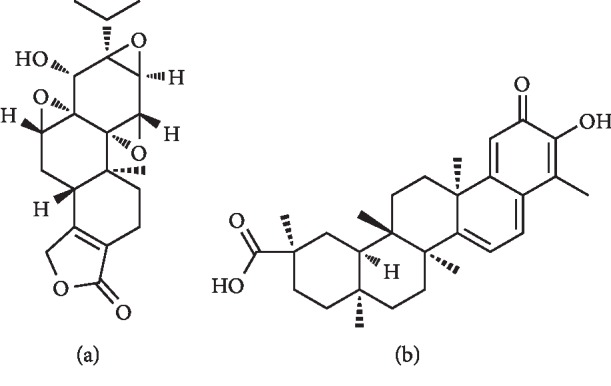
Chemical structures of (a) triptolide (CAS: 38748-32-2) and (b) celastrol (CAS: 34157-83-0).

The results of TCMSP retrieval, STITCH prediction, and SwissTargetPrediction prediction suggested that the 64 overlapping genes were linked to 17 compounds, and their interactions are listed in Supplementary [Supplementary-material supplementary-material-1]. The network of interactions between 64 overlapping genes and 17 compounds were constructed and visualized by Cytoscape and consisted of 81 nodes and 122 edges (Figure 3). The results exhibited that the 64 genes and 17 compounds were the related genes and active ingredients of THH against RA, respectively. As listed in Tables 1 and 2, the degree value of each gene or compound was used to identify their contribution difference to THH against RA. PTGS2, connected to 11 compounds, was selected as the hub gene of THH against RA. Triptolide and celastrol, connected to 27 genes and 23 genes, were considered as the key active ingredients of THH against RA. The chemical structures of triptolide and celastrol are shown in Figure 4. Additionally, the results of SystemsDock website indicated that the Docking Score values of PTGS2 with triptolide and celastrol were 7.10 and 6.84, respectively, suggesting that the combing degree of PTGS2 with triptolide or celastrol was very good.

### 3.4. Molecular Mechanisms of THH against RA

**Figure 5:**
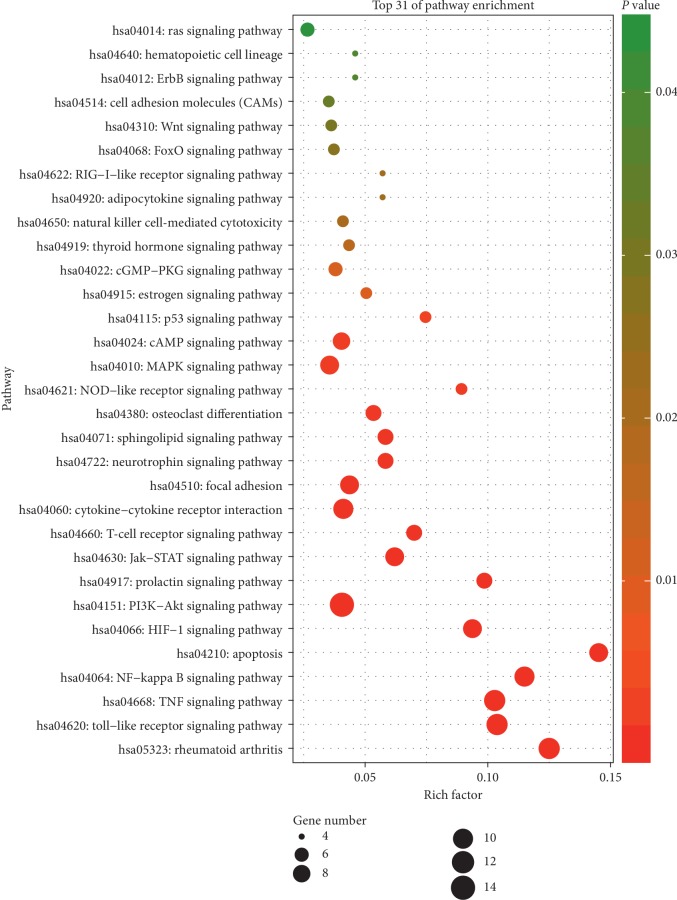
Bubble chart of 31 signaling pathways linked to THH against RA.

Pathway enrichment analysis for 64 overlapping genes was carried out on DAVID, and the results of KEGG pathway enrichment analysis suggested that 64 overlapping genes were significantly enrichment in 78 signaling pathways (*p* < 0.05). The 31 signaling pathways (Figure 5, Supplementary [Supplementary-material supplementary-material-1]) were directly linked to RA initiation and progression based on extensive literature retrieval, suggesting that these signaling pathways might be the molecular mechanisms of THH against RA. The hub gene PTGS2 of THH against RA was enriched in TNF signaling pathway and NF-kappa B signaling pathway. KEGG official website shows that the role of PTGS2 in the two signaling pathways is to regulate inflammation response, indicating that the key molecular mechanism of THH against RA might be to regulate inflammation response by interfering TNF and NF-kappa B signaling pathways.

## 4. Discussion

RA is characterized by persistent synovitis, systemic inflammation, and autoantibodies (particularly to rheumatoid factor and citrullinated peptide) [20]. RA affects about 1% of the population and results in a huge financial and emotional burden for both the individual and society [21]. Increased mortality in RA is widely recognized [22], so it is very meaningful to develop safe and effective anti-RA drugs. TCM plays an irreplaceable role in clinical treatment of RA in China [23]. However, the active ingredients and molecular mechanisms of TCM against RA remain largely unclear, and THH is one of them. In this work, network pharmacology was employed to resolve this issues, and the results of the present study illuminated the active ingredients and molecular mechanisms of THH against RA based on holistic perspective, the characteristic of TCM.

Absorption, distribution, metabolism, excretion, and toxicity (ADMET) evaluation of compounds is a common processing method to screen compounds in network pharmacology. ADMET property of one compound may affect ADMET property of another compound [24, 25], but ADMET evaluation ignores the possibility. TCM is a complex of many compounds, and ADMET evaluation of these compounds may remove some potential active ingredients. Therefore, ADMET evaluation was not employed to screen the compounds from THH to avoid the removal of some potential active ingredients of THH against RA in the present study. The genes-compounds network exhibited that the related active ingredients of THH against RA consisted of 17 compounds. The degree value of 17 compounds indicated that their contribution to the therapeutic effect of THH on RA were different, and triptolide and celastrol were the key active ingredients of THH against RA. Many studies indicated that triptolide showed well anti-RA effect, and the molecular mechanisms were related to many respects such as immunosuppression, inhibition of inflammation, induction of apoptosis, inhibition of angiogenesis, and protection of cartilage [26–30]. It was reported that celastrol regulated OPG/RANKL axis and inhibited the expressions of chemokine and inflammation cytokines in RA synovioblast, indicating that celastrol showed an important role in suppression of inflammation and bone erosion in RA [31]. These reports confirmed the correctness of network pharmacology-based prediction of the key active ingredients of THH against RA.

The genes-compounds network indicated that the related genes of THH against RA consisted of 64 genes. The results of pathway enrichment analysis for these genes showed that 31 signaling pathways were directly related to RA initiation and progression, indicating that the molecular mechanisms of THH against RA were linked to the 31 signaling pathways. Based on the existing literatures, the relationships between top 5 of pathway enrichment and RA were briefly discussed as follows. KEGG official website indicated that inflammatory cell infiltration, angiogenesis, inflammation, synovial pannus formation, joint destruction, and bone resorption are the key biological effects involved in rheumatoid arthritis signaling pathway, and these biological effects were directly related to RA pathogenesis [32–34]. It was reported that toll-like receptor was a potent driving force behind RA, and toll-like receptor signaling pathway played an important role in RA initiation and progression [35, 36]. Previous studies indicated that synovial inflammatory cells were significantly decreased after the anti-TNF-*α* mAb treatment, suggesting that TNF-*α* played an important role in RA pathogenesis [37–39]. The key pathogenesis of RA was overexpressed inflammatory cytokines and tissue injury mediated by persistent NF-kappa B activation, and agents could alleviate RA phenotype by blockade of NF-kappa B activation [40, 41]. Recent work indicated that the insufficient apoptosis of inflammatory cells in the RA joint might contribute to pathogenesis, and induction of inflammatory cells apoptosis is a feasible strategy for treating RA [42]. These reports confirmed the correctness of network pharmacology-based prediction of the molecular mechanisms of THH against RA. The relationships between other 26 signaling pathways and RA were not discussed in detail in this work, but their relationships could be easily identified by retrieving literatures.

The degree value of 64 genes indicated that their contribution to the therapeutic effect of THH on RA were different, and PTGS2 was the hub gene of THH against RA. In the 31 signaling pathways, PTGS2 was enriched in TNF and NF-kappa B signaling pathways, and KEGG official website shows that the role of PTGS2 in the two signaling pathways is to regulate inflammation response. Studies indicated that COX-2 enzyme, generated by PTGS2 gene, was selectively induced by proinflammatory cytokines at the site of inflammation to promote inflammation progression, and expression of the inducible COX-2 enzyme was selectively blocked by the potent anti-inflammatory drug dexamethasone [43]. Inflammation is the key driving factor to trigger RA clinical symptoms, such as joint damage, disability, and comorbidity, so anti-inflammation is a main therapeutic strategy [44]. It was reported that triptolide inhibited the IL-1*α*-induced production of PGE2 by selectively suppressing the gene expression and production of COX-2 in human synovial fibroblasts and suppressed the expression of TNF-*α* in synovia of collagen-induced RA rat and the expression and activity of NF-kappa B in synovium of collagen-induced RA rats [45, 46]. Celastrol could modulate inflammation through inhibition of the COX-2 activity and markedly alleviated the clinical signs, synovial hyperplasia, and inflammatory cell infiltration of joints in a collagen-induced RA rat model, related to inhibition of NF-kappa B activation [47–49]. Additionally, celastrol could inhibit the expression of TNF-*α* mRNA in human rheumatoid synoviocyte MH7A [31]. Based on these existing reports, it is reasonable to conclude that the key molecular mechanism of THH against RA was related to inhibition of inflammation response through inactivating TNF and NF-kappa B signaling pathways.

## 5. Conclusions

This work firstly explored the active ingredients and molecular mechanisms of THH against RA based on holistic perspective, the characteristic of TCM, with the aid of network pharmacology. The active ingredients of THH against RA consisted of 17 compounds, and triptolide and celastrol were the key active ingredients. The related genes of THH against RA included 64 target genes, and PTGS2 was the hub gene. The molecular mechanisms of THH against RA comprised 31 signaling pathways, and the key molecular mechanism was related to inhibition of inflammation response through inactivating TNF and NF-kappa B signaling pathways. In addition, this work provided scientific evidence to support the therapeutic effect of THH on RA.

## Figures and Tables

**Table 1:** Degree value of 64 genes in network.

No.	Gene	Value

1	XIAP	1
2	VTCN1	1
3	TP53	1
4	TNFRSF10A	1
5	TIMP2	1
6	STAT3	1
7	STAT1	1
8	SENP1	1
9	RORC	1
10	RELA	1
11	PTPN2	1
12	PDE4D	1
13	NFE2L2	1
14	NAMPT	1
15	MYC	1
16	MMP9	1
17	MCL1	1
18	KDR	1
19	KCNMA1	1
20	IL6	1
21	IL4	1
22	IL23A	1
23	IL2	1
24	IFNG	1
25	ICAM1	1
26	HSPA4	1
27	FOS	1
28	FN1	1
29	FLT1	1
30	F2	1
31	ESR1	1
32	CXCR4	1
33	CXCL8	1
34	CFLAR	1
35	CDKN1A	1
36	CD80	1
37	CD40	1
38	CD274	1
39	CD14	1
40	CCND1	1
41	CALM1	1
42	C3	1
43	BIRC5	1
44	BAX	1
45	AKR1B10	1
46	AKR1B1	1
47	ACP1	1
48	VEGFA	2
49	TNF	2
50	MAPK8	2
51	JUN	2
52	HSD11B1	2
53	HMOX1	2
54	CASP3	2
55	BCL2	2
56	ABCB1	2
57	PDE3A	3
58	PTGS1	4
59	OPRD1	7
60	NR3C1	7
61	ADRA1A	7
62	CA2	9
63	ADRB2	9
64	PTGS2	11

**Table 2:** Degree value of 17 compounds in network.

No.	Compound	Value
1	Hypodiolide A	1
2	Populnilic acid	1
3	Celafurine	1
4	3-Pyridinecarboxylic acid	1
5	1*β*,9*α*-Dibenzoyloxy-4-hydroxy-6*α*-nicotinoyloxy-*β*-dihydroagarofuran	1
6	Triptoditerpenic acid	4
7	Neotriptophenolide	6
8	Triptophenolide	6
9	Triptonoterpenol	6
10	Triptonoterpene	6
11	Triptonolide	6
12	Triptinin B	6
13	Triptonoterpene methyl ether	8
14	Triptoditerpenic acid B	8
15	Oleanolic acid	11
16	Celastrol	23
17	Triptolide	27

## Data Availability

The data used to support the findings of this study are available from the corresponding author upon request.

## Supplementary Materials

Supplementary Materials Supplementary material file includes 5 tables. Table S1: a list of 123 alkaloids and terpenoids from THH. Table S2: a list of 110 genes linked to 18 compounds from 123 alkaloids and terpenoids. Table S3: a list of 1871 genes linked to RA. Table S4: a list of the interactions between 17 compounds from THH and RA-related 64 target genes. Table S5: target genes in 31 signaling pathways related to THH against RA.
